# Two are better than one: the combinations of *Beauveria bassiana*, diatomaceous earth, and indoxacarb as effective wheat protectants

**DOI:** 10.1007/s11356-022-25075-1

**Published:** 2023-01-14

**Authors:** Waqas Wakil, Nickolas G. Kavallieratos, Erifili P. Nika, Abid Ali, Taha Yaseen, Muhammad Asrar

**Affiliations:** 1 Department of Entomology, University of Agriculture, Faisalabad, 38040 Pakistan; 2 Senckenberg German Entomological Institute, Eberswalder str 90, 15374 Müncheberg, Germany; 3 Laboratory of Agricultural Zoology and Entomology, Department of Crop Science, Agricultural University of Athens, 75 Iera Odos str, 11855 Athens, Attica Greece; 4 Department of Zoology, Government College University, Faisalabad, 38040 Pakistan

**Keywords:** Stored-product Coleoptera, Entomopathogenic fungus, Natural insecticide, Oxadiazine, Efficacy, Persistence, Grain

## Abstract

The current study evaluates the efficacy of the entomopathogenic fungus *Beauveria bassiana* (Balsamo-Crivelli) Vuillemin (Hypocreales: Cordycipitaceae), diatomaceous earth (DE) (Protect-It), and the oxadiazine indoxacarb, at single or combined applications on wheat kernels, for the management of the rusty grain beetle, *Cryptolestes ferrugineus* (Stephens) (Coleoptera: Laemophloeidae), the red flour beetle, *Tribolium castaneum* (Herbst) (Coleoptera: Tenebrionidae), the khapra beetle, *Trogoderma granarium* Everts (Coleoptera: Dermestidae), and the lesser grain borer, *Rhyzopertha dominica* (F.) (Coleoptera: Bostrychidae). The study was conducted between November 2020 and August 2021 in Faisalabad under a complete randomized block design. The combination of DE + indoxacarb was the most efficient as it caused higher overall mortalities ranging between 59.34 and 100%, and lower overall progeny production ranging between 8.35 and 33.70 individuals per vial, than all other treatments. *Beauveria bassiana* alone exhibited the lowest mortality rates ranging between 22.33 and 47.76%, and the highest offspring emergence, ranging between 51.33 and 78.55 individuals per vial. Similar pattern was observed when persistence bioassays were conducted. For a period of 120 days, the DE + indoxacarb was the most powerful combination against all tested species, providing overall mortality rates between 17.06 and 63.80%. The overall progeny production was lower for the insect individuals exposed on wheat treated with the DE + indoxacarb combination, ranging between 13.66 and 52.23 individuals per vial, and higher for those exposed to *B. bassiana* alone, ranging between 44.03 and 107.67 individuals per vial, for the entire duration of storage. However, the efficacy of all treatments decreased gradually during the course of storage. The findings of the current study indicate that the combinations of entomopathogenic fungi, DE, and indoxacarb can be used for the prolonged protection of stored wheat from the tested noxious insect species of stored products. Further research, which will include other inert dusts in combination with entomopathogenic fungi and indoxacarb, may provide additional knowledge towards an effective management of noxious species occurring in storages.

## Introduction

The rusty grain beetle, *Cryptolestes ferrugineus* (Stephens) (Coleoptera: Laemophloeidae), the red flour beetle, *Tribolium castaneum* (Herbst) (Coleoptera: Tenebrionidae), the khapra beetle, *Trogoderma granarium* Everts (Coleoptera: Dermestidae), and the lesser grain borer, *Rhyzopertha dominica* (F.) (Coleoptera: Bostrychidae) are pests infesting a wide variety of commodities and products, stored in facilities like mills, grain bins, silos, warehouses, cargo containers, and retail stores (Hagstrum and Subramanyam 2009; Edde 2012; Myers and Hagstrum 2012; Opit et al. 2012; EPPO 2013, 2022; Majeed et al. 2015; Athanassiou et al. 2019). These species cause quantitative and qualitative damage to the infested stored products, ranging between 20 and 40% of the annual global crop yield (Hill 2003; Arthur et al. 2012; FAO 2022). *Trogoderma granarium* and *R.* *dominica* are primary pests that initiate the infestation, while *C. ferrugineus* and *T. castaneum* are secondary pests that continue the losses of the already damaged kernels (Hill 2003; Rees 2004; Robinson 2005; Hagstrum and Subramanyam 2009; Kumar 2017; Athanassiou et al. 2019; Galecki et al. 2019). All aforementioned species are serious pests in storages of Pakistan (Wakil and Schmitt 2015; Wakil et al. 2021a).

Entomopathogenic fungi are effective eco-friendly alternatives, safe for humans and the environment (Khashaveh et al. 2011; Flinn and Schöller 2012; Wakil and Schmitt 2015; Batta and Kavallieratos 2018; Schöller et al. 2018; Wakil et al. 2021b). The mode of action of entomopathogenic fungi relies to the attachment of the fungal spores onto the insects’ cuticle and their sequent germination and penetration inside the host (Zimmermann 2007). This results in the deterioration of the immune system of insects, fungal growth, and the emergence of new fungal conidia from the dead bodies of insects to infest new individuals (Zimmermann 2007; Dannon et al. 2020). Among the most common entomopathogenic fungi species is *Beauveria bassiana* (Balsamo-Crivelli) Vuillemin (Hypocreales: Cordycipitaceae), which has been previously examined against several stored-product insects such as the sawtoothed grain beetle, *Oryzaephilus surinamensis* (L.) (Coleoptera: Silvanidae), *Liposcelis paeta* (Pearman) (Psocoptera: Liposcelididae), the rice weevil, *Sitophilus oryzae* (L.) (Coleoptera: Curculionidae), the granary weevil, *Sitophilus granarius* (L.) (Coleoptera: Curculionidae), *C. ferrugineus*, *T. castaneum*, *T. granarium*, and *R. dominica* (Athanassiou et al. 2007; El-Sebai 2011; Shafighi et al. 2014; Akmal et al. 2017; Latifian et al. 2018; Rehman et al 2019; Wakil et al. 2021b, c, d).

The performance of entomopathogenic fungi can be enhanced when combined with other synthetic or natural insecticides and beneficial organisms (Athanassiou et al. 2007; Batta and Kavallieratos 2018; Wakil et al. 2021b, c, d). Natural insecticides that have been used for this purpose are diatomaceous earths (DEs) (Batta and Kavallieratos 2018), generated by unicellular plants like algae fossils, which are silicon dioxide consisted inert dusts (Korunic 1998, 2013; Losic and Korunic 2018). Their mode of action relies on the friction between the DEs and the cuticle of insects, which causes discontinuities on the waxy external cuticle layer, since DEs absorb lipids (Korunic 1998; Losic and Korunic 2018). As an outcome, the insect individuals dehydrate and die due to desiccation (Korunic 1998; Subramanyam and Roesli 2000; Mewis and Ulrichs 2001; Losic and Korunic 2018). Numerous DEs have been used for the management of different developmental stages of stored-product pests, e.g., the confused flour beetle, *Tribolium confusum* Jacquelin du Val (Coleoptera: Tenebrionidae), the yellow mealworm beetle, *Tenebrio molitor* L. (Coleoptera: Tenebrionidae), the Indianmeal moth, *Plodia interpunctella* (Hübner) (Lepidoptera: Pyralidae), the Mediterranean flour moth, *Ephestia kuehniella* Zeller (Lepidoptera: Pyralidae), *S. oryzae*, *S. granarius*, *C. ferrugineus*, *T. castaneum*, *L. paeta*, *T. granarium*, and *R. dominica* as surface and grain insecticides (Mewis and Ulrichs 2001; Mahdneshin et al. 2009; Nwaubani et al. 2014; Shafighi et al. 2014; Wakil and Schmitt 2015; Kavallieratos et al. 2019; Rizwan et al. 2019; Wakil et al. 2021b, c, d, 2022).

Indoxacarb belongs to oxadiazine insecticides that block sodium channels causing hyperpolarization to the cellular membranes (Wing et al. 2000). Previous studies have reported that indoxacarb is efficient for the management of insects belonging to Lepidoptera, Coleoptera, Diptera, Homoptera, and Blatttodea (Wing et al. 2000; Liu et al. 2002; Buczkowski et al. 2008; Gondhalekar et al. 2010; Afzal and Shad 2016; Zahn et al. 2019; Oliveira et al. 2021). Concerning its performance against stored-product insects, indoxacarb exhibited variable efficacy against 13 populations of the maize weevil, *Sitophilus zeamais* (Motschulsky) (Coleoptera: Curculionidae) (Haddi et al. 2015). By applying different doses on maize kernels, the authors estimated the LD_50_ values and the behavioral alterations of the exposed individuals. One other study revealed that indoxacarb was efficient against *S. oryzae* and *R.* *dominica* when they were exposed on maize and wheat (Miliordos et al. 2017). In a recent study, Khan (2020) calculated the LD_50_ and LD_99_ of indoxacarb against 7 strains of *R.* *dominica*, *S. zeamais*, *S. oryzae*, *T. castaneum*, and *O. surinamensis*. The results varied among strains, when the insecticide was treated on wheat, 7 days post-exposure.

Through an extended search of the published literature, even though different *B. bassiana* isolates and DE formulations have been used alone, in combination, or in combination with chemical insecticides, their insecticidal activity with indoxacarb has yet to be evaluated since there are no available data. Thus, the objectives of this study are to assess for the first time the efficacy and persistence of binary combinations of indoxacarb with *B. bassiana* and DE against adults of *C. ferrugineus*, *T. castaneum*, *T. granarium*, and *R. dominica* on infested wheat, in terms of mortality and progeny production.

## Materials and methods

### Insects

The unsexed *C. ferrugineus*, *T. castaneum*, *T. granarium*, and *R.* *dominica* adult individuals were acquired from local storage facilities of Faisalabad and reared since 2009 at the Department of Entomology, University of Agriculture Faisalabad Pakistan. The rearing medium for the development of *C. ferrugineus* was wheat flour, for *T. castaneum* was wheat flour with extra 5% brewer’s yeast, and for *R. dominica* and *T. granarium* was wheat, at 30 °C, 65% relative humidity (RH), and total absence of light (Ganesan et al. 2021; Wakil et al. 2021b). The tested individuals of *R.* *dominica*, *T. castaneum*, and *C. ferrugineus* were < 2 weeks old, whereas the tested individuals of *T. granarium* were < 24 h old (Wakil and Schmitt 2015; Wakil et al. 2021b).

### Commodity

Soft wheat, *Triticum aestivum* L. (var. Lasani-2008) without pests, debris, and pesticides was used in the bioassays. The kernel moisture content was calculated at 12.8% with the utilization of a moisture meter (Dickey-John Multigrain CAC II; Dickey-John Co., USA).

### Entomopathogenic fungus culture

The inoculation of *B. bassiana* was conducted in Petri dishes (100 mm) covered with Sabouraud Dextrose Agar (SDA), wrapped with a piece of parafilm. Dishes were incubated at 25 °C, 70% RH, and 14 light:10 dark h photoperiod in a MIR-254 incubator (Panasonic, Japan), for a period of 10 days (Usman et al. 2020). Fungal conidia were collected with the utilization of a sterile scalpel from the SDA surface and then conveyed in a 50 ml conical tube containing 30 ml of sterile 0.05% Tween 80 (Merck, Kenilworth, NJ, USA) solution. Thereinafter, the suspension of conidia was agitated with a vortex (Classic Vortex Mixer, Velp Scientifica Srl, Usmate Velate, Italy) for 5 min, along with eight sterile glass beads to aim the agitation. The determination of the conidia concentration (1 × 10^7^ conidia/ml suspension) was conducted with a Neubauer-improved hemocytometer (Marienfeld, Lauda-Königshofen, Germany) under a microscope. The germination of conidia was taken place by the inoculation of a 0.1 ml solution containing 1 × 10^6^ conidia/ml, on two 60 mm dishes with SDA + Yeast (SDAY). Then, these dishes were covered with parafilm and incubated for 16 h, set at 25 °C and 14 light:10 dark h. After incubation, dishes were covered with a cover slip that was sterilized. In each dish, 200 conidia were counted and regarded germinated when conidia < germ tube (Inglis et al. 2012; Usman et al. 2020; Gulzar et al. 2021). The countings were conducted under a microscope (Euromex BB.1152-PLi, Euromex Microscopen bv, Arnhem, The Netherlands) with 400× total magnification. Before the beginning of the bioassays, > 94% of conidia was viable.

### Formulation

The formulation used in the bioassays was Steward 150 EC. This product contains 16.5% indoxacarb active ingredient (a.i.) (FMC United Pvt. Ltd., Lahore, Punjab, Pakistan).

### Diatomaceous earth

The DE formulation Protect-It (Hedley Technologies Inc., Mississauga, Ontario, Canada) was used in the bioassays. This DE originates from freshwater, containing < 0.9% total crystalline silica content, 10% silica gel, and 90% amorphous silicon dioxide (Wakil et al. 2021d).

### Laboratory bioassays

For the bioassays, six treatments and control were applied on wheat. The treatments were *B. bassiana* alone at 1 × 10^7^ conidia/kg wheat (Bb), indoxacarb alone at 5 ppm (5 mg a.i./kg wheat), DE alone at 150 ppm (150 mg DE/kg wheat), *B. bassiana* + indoxacarb combination, *B. bassiana* + DE combination, indoxacarb + DE combination, and control (0.05% Tween 80). Wheat lots of 1 kg were laid in slim layers on separate trays for each treatment. The *B. bassiana*, indoxacarb, and the control solutions were applied on the wheat lots as liquids with a separate airbrush (Master Multipurpose Airbrush, USA), while the DE formulation was applied as dust. The wheat portions were sprayed using 1 ml of the suspension of conidia or with 1 ml of aqueous solution containing the analogous volume of indoxacarb. Thereafter, the treated wheat portions were conveyed to separate 3-l glass containers for a manual shake of 10 min total duration, to assure the equal distribution of each killing agent. As far as the combinations are concerned, the first application was *B. bassiana* followed by the DE (Bb + DE). In the combinations with indoxacarb, this insecticide was applied first and then the conidia of *B. bassiana* (Bb + indoxacarb) or the DE (DE + indoxacarb). Subsequently, the treated portions of wheat were shaken as above in different 3-l containers. From each treated wheat lot, three samples of 100 g were weighted with a balance (ELB 300 Shimadzu compact balance, Kyoto, Japan) on separate layers. The weighted samples were put into plastic containers of 11 cm height and 6.5 cm diameter. Polytetrafluoroethylene (60 wt % dispersion in water) (Sigma-Aldrich Chemie GmbH, Taufkirchen, Germany) was applied on the inside necks of the plastic containers to impede the insects from escaping. Next, fifty *C. ferrugineus* adults were put into each plastic vial. The adequate aeration of these vials was achieved by cyclic holes of 1.5 cm diameter on the lids, cladded with muslin cloth. The prepared vials were incubated at 30 °C and 65% RH for 7 and 14 days to assess the mortality with a stereomicroscope (Leica Wild M3B, Heerbrugg, Switzerland) under 40× total magnification. For each exposure interval, we prepared new series of vials. Upon the end of the 14-day experimental period, all tested insects were separated from the wheat lots. Afterwards, wheat was returned into the vials and into the incubators at the aforementioned abiotic conditions to document progeny production. All the above procedure was copied three more times with new vials, wheat portions, and insects. The same process was replicated for *T. castaneum*, *T. granarium*, and *R.* *dominica*. The emergence of offspring was documented after 62 days (Wakil et al. 2021c), 46 days (Kavallieratos et al. 2017a), and 62 days (Wakil et al. 2021c) for *T. castaneum*, *T. granarium*, and *R. dominica*, respectively. Progeny of *R. dominica* was adults as the immature stages of this species are into the grains (Aitken 1975), while for all the other species were adults and their immature stages. The bioassays were conducted between November 2020 and August 2021.

### Persistence bioassays

The insecticidal effects of *B. bassiana*, DE, and indoxacarb, their binary combinations (Bb + DE, Bb + indoxacarb, and DE + indoxacarb), and control were recorded for a period of 120 days, 7 or 14 days after the initial exposure, against *C. ferrugineus*, *T. castaneum*, *T. granarium*, and *R. dominica*. Every 30 days, mortalities of the exposed individuals of the aforementioned species and the offspring emergence were estimated as in the laboratory bioassays. The wheat lots were kept in glass containers in incubators set at 30 °C and 65% RH, for the whole duration of the experiment.

### Statistical analysis

#### Laboratory bioassays

Mortality values were corrected by Abbott’s formula (Abbott 1925). Data were transformed to log (x + 1) before the analysis to make the variance normal (Zar 2014; Scheff and Arthur 2018). Data for each species were analyzed with a two-way analysis of variance (ANOVA) with treatment and exposure interval being the main effects, while the response variable was mortality. Interactions of the main effects were taken into consideration in the analysis. Similarly, for the progeny production, data were analyzed with a two-way ANOVA, having treatment and species as main effects, while the response variable was the number of offspring. Interactions of the main effects were incorporated into the analysis. Minitab 17 software (Minitab 2010) was utilized for all the conducted analyses. To compare mortality and progeny means, the Tukey-Kramer (HSD) test at 5% importance was used (Sokal and Rohlf 1995).

#### Persistence bioassays

Mortality values were corrected by Abbott’s formula (Abbott 1925). Data were transformed to log (x + 1) before the analysis to make the variance normal (Zar 2014; Scheff and Arthur 2018). Data for each species were analyzed with a two-way ANOVA with treatment and period of storage being the main effects, while the response variable was mortality. Interactions of the main effects were taken into consideration in the analysis. The two-way ANOVA was conducted separately for each species and initial exposure (7 or 14 days). For the progeny production, data were analyzed with a two-way ANOVA, having treatment and period of storage as main effects, while the response variable was the number of offspring, per species. Interactions of the main effects were incorporated into the analysis. Minitab 17 software (Minitab 2010) was utilized for all the conducted analyses. To compare mortality and progeny means, the Tukey-Kramer (HSD) test at 5% importance was used (Sokal and Rohlf 1995).

## Results

### Mortality and progeny in the laboratory

Concerning *C. ferrugineus*, main effects and their interaction were found significant (Table 1). The lowest mortality rates were observed for the individuals exposed on wheat treated with *B. bassiana*, not exceeding 31.21 and 43.08%, 7 and 14 days post-exposure, respectively (Table 2). The combination of DE + indoxacarb caused the highest mortality (93.88%) among all other tested insecticidal treatments, followed by Bb + indoxacarb (82.35%) and Bb + DE (71.47%), after 14 days of the initial exposure. Regarding *T. castaneum*, main effects and their interaction were significant (Table 1). The most efficient treatment was the combination of DE + indoxacarb that caused 66.13 and 87.42% mortality, while the least efficient was *B. bassiana* alone causing 28.46 and 39.65% mortality, after 7 and 14 days of initial exposure respectively (Table 2). As far as *T. granarium* is concerned, main effects and their interaction were significant (Table 1). When the insecticides were tested alone, mortality rates ranged between 22.33 (*B. bassiana*) and 33.52% (indoxacarb) after 7 days of exposure and between 31.86 (*B. bassiana*) and 47.76% (indoxacarb) after 14 days of initial exposure (Table 2). The mortality levels of the combinations were higher than the insecticides alone, with the treatment of DE + indoxacarb combination exhibiting the highest rates (59.34 and 81.67%, 7 and 14 days post-exposure, respectively), followed by Bb + indoxacarb (52.23 and 74.52%, after 7 and 14 days of exposure, respectively) and Bb + DE (40.67 and 56.26%, 7 and 14 days post-exposure, respectively). Main effects and their interaction were significant in the case of *R.* *dominica* (Table 1). Complete (100.00%) mortality was achieved for the adults exposed on the combination of DE + indoxacarb 14 days post-exposure, while 73.58% of the exposed *R.* *dominica* were dead after 7 days of initial exposure to the same combination (Table 2). When *B. bassiana* was tested alone, mortality levels were the lowest, not exceeding 38.63 and 47.42%, after 7 and 14 days of exposure. The combination of Bb + indoxacarb was the second most efficient insecticidal combination that killed 65.77 and 92.18% of the exposed *R.* *dominica* individuals, after 7 and 14 days.

**Table 1:** ANOVA parameters of main effects and associated interaction for mortality levels of *C. ferrugineus*, *T. castaneum*, *T. granarium*, and *R. dominica* adults under laboratory trials (total *df* = 71 for all species)

Species		*C. ferrugineus*		*T. castaneum*		*T. granarium*		*R. dominica*	
Source	*df*	*F*	*P*	*F*	*P*	*F*	*P*	*F*	*P*
Treatment	5	168.85	< 0.01	126.49	< 0.01	107.91	< 0.01	200.66	< 0.01
Exposure interval	1	232.32	< 0.01	163.63	< 0.01	139.14	< 0.01	372.59	< 0.01
Treatment × exposure interval	5	2.95	0.01	2.82	0.02	2.82	0.02	9.34	< 0.01

**Table 2:** Mean mortality (% ± SE) of *C. ferrugineus*, *T. castaneum*, *T. granarium*, and *R. dominica* adults exposed for 7 and 14 days on wheat treated with one dose of *B. bassiana* (Bb) (1 × 10^7^ conidia/kg wheat), one dose of DE (150 ppm), one dose of indoxacarb (5 ppm), and their combinations (Bb + DE, Bb + indoxacarb, and DE + indoxacarb) in laboratory trials

Species	Treatment	7 days	14 days	*F*	*P*
*C. ferrugineus*	Bb	31.21 ± 1.27 Bc	43.08 ± 2.16 Ad	22.3	< 0.01
	DE	37.56 ± 2.30 Bc	48.45 ± 1.00 Ad	18.7	< 0.01
	Indoxacarb	48.47 ± 1.98 Bb	65.10 ± 1.67 Ac	41.0	< 0.01
	Bb + DE	52.23 ± 1.48 Bb	71.47 ± 2.73 Ac	38.2	< 0.01
	Bb + indoxacarb	64.43 ± 2.35 Ba	82.35 ± 2.07 Ab	32.7	< 0.01
	DE + indoxacarb	70.88 ± 1.50 Ba	93.88 ± 1.19 Aa	142	< 0.01
	*F*	66.3	105		
	*P*	< 0.01	< 0.01		
*T. castaneum*	Bb	28.46 ± 1.44 Be	39.65 ± 1.50 Ad	28.8	< 0.01
	DE	34.84 ± 3.01 Bde	43.34 ± 2.05 Ad	6.67	0.02
	Indoxacarb	40.29 ± 2.25 Bcd	55.60 ± 1.97 Ac	26.0	< 0.01
	Bb + DE	48.80 ± 1.75 Bbc	63.03 ± 1.67 Ac	34.5	< 0.01
	Bb + indoxacarb	57.30 ± 2.50 Bab	78.63 ± 1.26 Ab	57.8	< 0.01
	DE + indoxacarb	66.13 ± 2.49 Ba	87.42 ± 2.45 Aa	36.9	< 0.01
	*F*	38.7	104		
	*P*	< 0.01	< 0.01		
*T. granarium*	Bb	22.33 ± 2.56 Bd	31.86 ± 1.36 Ad	10.8	< 0.01
	DE	27.76 ± 3.08 Acd	38.55 ± 2.00 Acd	4.55	0.05
	Indoxacarb	33.52 ± 1.89 Bbc	47.76 ± 2.11 Abc	25.1	< 0.01
	Bb + DE	40.67 ± 1.73 Bb	56.26 ± 1.90 Ab	36.5	< 0.01
	Bb + indoxacarb	52.23 ± 2.04 Ba	74.52 ± 2.83 Aa	36.3	< 0.01
	DE + indoxacarb	59.34 ± 0.94 Ba	81.67 ± 1.09 Aa	239	< 0.01
	*F*	44.2	63.8		
	*P*	< 0.01	< 0.01		
*R. dominica*	Bb	38.63 ± 1.66 Bd	47.42 ± 1.43 Ad	16.0	< 0.01
	DE	41.33 ± 1.16 Bd	53.21 ± 1.98 Ad	26.6	< 0.01
	Indoxacarb	52.17 ± 1.45 Bc	71.13 ± 2.59 Ac	40.7	< 0.01
	Bb + DE	54.24 ± 1.64 Bc	76.27 ± 1.91 Ac	75.9	< 0.01
	Bb + indoxacarb	65.77 ± 1.10 Bb	92.18 ± 1.71 Ab	167	< 0.01
	DE + indoxacarb	73.58 ± 2.37 Ba	100.00 ± 0.00 Aa	124	< 0.01
	*F*	69.9	134		
	*P*	< 0.01	< 0.01		

For each species, within each treatment, means followed by the same uppercase letter are not significantly different; *df* = 1, 11, Tukey–Kramer (HSD) test at *P* = 0.05. For each species, within each exposure interval, means followed by the same lowercase letter are not significantly different; *df* = 5, 35, Tukey–Kramer (HSD) test at *P* = 0.05

Regarding progeny production of all the tested species, the main effects and their interaction were significant (Table 3). The DE + indoxacarb combination was the most successful towards all tested insect species, ranging from 8.35 to 33.70 individuals, and *B. bassiana* alone was the least efficient for controlling the progeny production, ranging from 51.33 to 78.55 individuals (Table 4). All tested insecticidal treatments caused significant reduction to the progeny production in comparison with control. *Rhyzopertha* *dominica* had the lowest offspring emergence while *T. granarium* had the highest progeny at all tested insecticidal treatments.

**Table 3:** ANOVA parameters of main effects and associated interaction for progeny production of *C. ferrugineus*, *T. castaneum*, *T. granarium*, and *R. dominica* individuals in laboratory trials (total *df* = 167 for all species)

Source	*df*	*F*	*P*
Treatment	6	2541.71	< 0.01
Species	3	551.28	< 0.01
Treatment × species	18	11.78	< 0.01

**Table 4:** Mean number (± SE) of *C. ferrugineus*, *T. castaneum*, *T. granarium*, and *R. dominica* individuals per vial following a 14-day exposure interval of parents on wheat treated with one dose of *B. bassiana* (Bb) (1 × 10^7^ conidia/kg wheat), one dose of DE (150 ppm), one dose of indoxacarb (5 ppm), their combinations (Bb + DE, Bb + indoxacarb, and DE + indoxacarb), and control in laboratory trials

Treatment	*C. ferrugineus*	*T. castaneum*	*T. granarium*	*R. dominica*
Bb	61.43 ± 0.73 b	65.58 ± 1.22 b	78.55 ± 1.49 b	51.33 ± 1.06 b
DE	52.01 ± 0.90 c	57.25 ± 1.83 c	67.21 ± 1.25 c	42.35 ± 0.57 c
Indoxacarb	45.71 ± 1.52 d	53.11 ± 1.32 c	64.40 ± 1.15 c	36.13 ± 1.11 d
Bb + DE	34.10 ± 0.38 e	39.70 ± 0.87 d	46.18 ± 1.04 d	22.43 ± 1.06 e
Bb + indoxacarb	21.63 ± 0.87 f	32.40 ± 0.82 e	39.23 ± 1.77 e	13.48 ± 0.77 f
DE + indoxacarb	15.26 ± 1.07 g	23.31 ± 1.15 f	33.70 ± 0.86 e	8.35 ± 0.57 g
Control	117.43 ± 1.05 a	101.12 ± 1.79 a	115.67 ± 1.63 a	95.48 ± 0.92 a
*F*	1185	370	432	1084
*P*	< 0.01	< 0.01	< 0.01	< 0.01

For each species, means followed by the same lowercase letter are not significantly different; *df* = 6, 41, Tukey–Kramer (HSD) test at *P* = 0.05

### Persistence in the laboratory

Regarding *C. ferrugineus*, main effects and their interaction were significant after 7 days (Table 5) and 14 days (Table 7) of initial exposure. The highest mortality was observed for the DE + indoxacarb combination at 0 day of trial, reaching 61.07% 7 days (Table 6) and 95.22% 14 days (Table 8) post-exposure. After 7 days of exposure, at the 120 days trial, indoxacarb exhibited the lowest mortality (8.53%), while the DE + indoxacarb combination caused the highest mortality 38.58% (Table 6). The *B. bassiana* alone had the lowest mortality rates during all trials, 14 days post-exposure (Table 8). The DE + indoxacarb combination was the most efficient at all trials, after 14 days of initial exposure.

**Table 5:** ANOVA parameters of main effects and associated interaction for mortality levels of *C. ferrugineus*, *T. castaneum*, *T. granarium*, and *R. dominica* adults after 7 days of exposure in laboratory persistence trials (total *df* = 179 for all species)

Species		*C. ferrugineus*		*T. castaneum*		*T. granarium*		*R. dominica*	
Source	*df*	*F*	*P*	*F*	*P*	*F*	*P*	*F*	*P*
Treatment	5	278.76	< 0.01	199.52	< 0.01	203.45	< 0.01	323.34	< 0.01
Period of storage	4	2660.35	< 0.01	186.99	< 0.01	153.49	< 0.01	196.95	< 0.01
Treatment × period of storage	20	3.04	< 0.01	2.94	< 0.01	2.40	< 0.01	2.50	< 0.01

**Table 6:** Mean mortality (% ± SE) of *C. ferrugineus*, *T. castaneum*, *T. granarium*, and *R. dominica* adults exposed for 7 days on wheat treated with one dose of *B. bassiana* (Bb) (1 × 10^7^ conidia/kg wheat), one dose of DE (150 ppm), one dose of indoxacarb (5 ppm), and their combinations (Bb + DE, Bb + indoxacarb, and DE + indoxacarb) in five laboratory trials carried out from 0 to 120 days after treatment

Species	Treatment	0 days	30 days	60 days	90 days	120 days	*F*	*P*
*C. ferrugineus*	Bb	26.62 ± 1.75 Ad	21.50 ± 0.72 Bd	16.70 ± 1.19 BCd	12.62 ± 1.22 CDd	10.58 ± 0.97 De	28.7	< 0.01
	DE	35.15 ± 2.32 Ac	28.32 ± 2.53 ABcd	25.59 ± 2.01 Bc	21.51 ± 0.93 BCc	17.40 ± 0.86 Cd	13.0	< 0.01
	Indoxacarb	41.28 ± 1.00 Ac	34.12 ± 1.11 Bc	24.57 ± 1.66 Ccd	15.68 ± 1.22 Dd	8.53 ± 1.61 Ee	96.5	< 0.01
	Bb + DE	48.44 ± 1.76 Ab	42.65 ± 1.36 ABb	37.54 ± 0.84 BCb	32.41 ± 1.20 Cb	25.58 ± 1.68 Dc	39.3	< 0.01
	Bb + indoxacarb	52.57 ± 0.99 Ab	44.38 ± 1.69 Bb	39.24 ± 2.29 Bab	38.23 ± 1.09 Ba	31.73 ± 1.13 Cb	25.9	< 0.01
	DE + indoxacarb	61.07 ± 1.31 Aa	53.58 ± 1.70 Aa	45.71 ± 2.70 Ba	42.31 ± 1.58 Ba	38.58 ± 1.33 Ba	23.3	< 0.01
	*F*	58.0	51.7	33.3	100	84.0		
	*P*	< 0.01	< 0.01	< 0.01	< 0.01	< 0.01		
*T. castaneum*	Bb	22.18 ± 1.51 Ad	15.35 ± 1.14 Bd	13.63 ± 1.33 BCe	9.54 ± 1.35 CDd	6.13 ± 1.39 Dd	20.3	< 0.01
	DE	29.37 ± 2.61 Acd	26.62 ± 1.49 Ac	24.57 ± 1.39 Acd	17.05 ± 0.98 Bc	13.64 ± 1.53 Bc	15.4	< 0.01
	Indoxacarb	35.12 ± 1.99 Ac	28.30 ± 2.01 Ac	19.44 ± 1.11 Bde	12.27 ± 1.56 Ccd	10.23 ± 1.48 Ccd	40.1	< 0.01
	Bb + DE	47.42 ± 1.73 Ab	39.59 ± 0.84 Bb	31.73 ± 1.42 Cbc	25.62 ± 1.96 Cb	17.38 ± 1.33 Dbc	59.5	< 0.01
	Bb + indoxacarb	51.21 ± 1.41 Aab	42.31 ± 1.29 Bb	35.14 ± 3.53 BCab	28.67 ± 1.69 CDb	21.48 ± 1.69 Dab	30.5	< 0.01
	DE + indoxacarb	56.32 ± 1.19 Aa	51.20 ± 1.87 Aa	42.31 ± 2.24 Ba	36.51 ± 1.19 Ba	27.30 ± 2.56 Ca	37.1	< 0.01
	*F*	55.0	73.7	27.4	48.4	20.1		
	*P*	< 0.01	< 0.01	< 0.01	< 0.01	< 0.01		
*T. granarium*	Bb	12.64 ± 1.36 Ae	7.83 ± 1.61 ABd	5.44 ± 1.36 BCb	1.36 ± 0.86 CDd	0.00 ± 0.00 Dc	18.4	< 0.01
	DE	19.45 ± 1.13 Ade	15.70 ± 0.87 ABc	13.64 ± 1.11 Bb	8.52 ± 1.32 Cc	2.38 ± 0.62 Dc	40.4	< 0.01
	Indoxacarb	23.88 ± 1.62 Acd	18.07 ± 1.30 Bc	11.26 ± 0.88 Cb	3.42 ± 1.02 Dd	0.00 ± 0.00 Dc	79.7	< 0.01
	Bb + DE	31.40 ± 1.38 Abc	27.63 ± 1.22 ABb	24.58 ± 1.51 Ba	18.42 ± 1.56 Cb	11.60 ± 1.55 Db	28.9	< 0.01
	Bb + indoxacarb	37.19 ± 2.30 Ab	31.42 ± 2.98 ABb	26.63 ± 2.13 ABa	21.16 ± 0.88 BCab	15.68 ± 1.22 Cab	10.6	< 0.01
	DE + indoxacarb	46.40 ± 2.56 Aa	39.57 ± 1.03 Aa	31.74 ± 2.23 Ba	25.92 ± 1.09 Ba	17.06 ± 1.24 Ca	42.6	< 0.01
	*F*	46.5	48.8	22.2	76.7	64.1		
	*P*	< 0.01	< 0.01	< 0.01	< 0.01	< 0.01		
*R. dominica*	Bb	32.74 ± 2.05 Ad	27.63 ± 1.10 ABc	22.51 ± 1.45 BCd	18.41 ± 1.36 CDe	15.68 ± 1.77 Dd	18.9	< 0.01
	DE	38.57 ± 2.95 Acd	33.78 ± 2.25 ABc	28.32 ± 1.20 BCcd	23.90 ± 1.06 Cde	21.49 ± 1.13 Cd	13.9	< 0.01
	Indoxacarb	47.43 ± 0.90 Abc	42.66 ± 2.96 Ab	31.74 ± 0.90 Bc	25.23 ± 1.75 BCd	18.42 ± 1.03 Cd	49.5	< 0.01
	Bb + DE	53.21 ± 1.91 Ab	48.12 ± 1.06 ABb	44.70 ± 1.19 Bb	38.56 ± 0.77 Cc	29.36 ± 1.42 Dc	47.8	< 0.01
	Bb + indoxacarb	67.56 ± 2.46 Aa	61.42 ± 1.39 Aa	52.55 ± 2.41 Ba	45.40 ± 1.04 Bb	37.20 ± 0.97 Cb	46.5	< 0.01
	DE + indoxacarb	72.37 ± 1.49 Aa	64.47 ± 1.24 Ba	56.66 ± 2.58 BCa	51.86 ± 1.63 Ca	43.34 ± 1.61 Da	38.6	< 0.01
	*F*	57.3	63.7	63.3	103	65.5		
	*P*	< 0.01	< 0.01	< 0.01	< 0.01	< 0.01		

For each species, within in each treatment, means followed by the same uppercase letter are not significantly different; *df* = 4, 29, Tukey–Kramer (HSD) test at *P* = 0.05. For each species, within each period of storage, means followed by the same lowercase letter are not significantly different; *df* = 5, 35, Tukey–Kramer (HSD) test at *P* = 0.05

Main effects and their interaction regarding *T. castaneum* were significant 7 days (Table 5) and 14 days (Table 7) post-exposure. The highest and the lowest mortalities were noted for the DE + indoxacarb combination and *B. bassiana* alone treatments respectively, ranging between 27.30 and 56.32% (DE + indoxacarb) and 6.13 and 22.18% (*B. bassiana* alone) at all trials, 7 days post-exposure (Table 6). After 14 days of initial exposure, the DE + indoxacarb combination and *B. bassiana* alone treatments killed the most and least *T. castaneum* exposed adults at all trials (ranges: 43.67–84.31% and 13.31–27.65%, respectively) (Table 8).

**Table 7:** ANOVA parameters of main effects and associated interaction for mortalities of *C. ferrugineus*, *T. castaneum*, *T. granarium*, and *R. dominica* adults after 14 days of exposure in laboratory persistence trials (total *df* = 179 for all species)

		*C. ferrugineus*		*T. castaneum*		*T. granarium*		*R. dominica*	
Source	*df*	*F*	*P*	*F*	*P*	*F*	*P*	*F*	*P*
Treatment	5	832.45	< 0.01	508.98	< 0.01	345.50	< 0.01	549.11	< 0.01
Period of storage	4	350.89	< 0.01	198.88	< 0.01	214.60	< 0.01	295.07	< 0.01
Treatment × period of storage	20	5.28	< 0.01	6.12	< 0.01	8.84	< 0.01	2.87	< 0.01

**Table 8:** Mean mortality (% ± SE) of *C. ferrugineus*, *T. castaneum*, *T. granarium*, and *R. dominica* adults exposed for 14 days on wheat treated with one dose of *B. bassiana* (Bb) (1 × 10^7^ conidia/kg wheat), one dose of DE (150 ppm), one dose of indoxacarb (5 ppm), and their combinations (Bb + DE, Bb + indoxacarb, and DE + indoxacarb) in five laboratory trials carried out from 0 to 120 days after treatment

Species	Treatment	0 days	30 days	60 days	90 days	120 days	*F*	*P*
*C. ferrugineus*	Bb	38.23 ± 2.99 Ad	33.09 ± 2.42 ABd	25.58 ± 1.76 BCe	21.48 ± 1.92 Cd	18.75 ± 1.50 Cc	13.7	< 0.01
	DE	43.68 ± 1.71 Ad	36.16 ± 1.73 Bd	31.73 ± 0.85 BCd	26.29 ± 1.83 CDcd	21.49 ± 1.13 Dc	32.6	< 0.01
	Indoxacarb	56.66 ± 1.39 Ac	47.44 ± 1.43 Bc	38.57 ± 1.05 Cc	29.67 ± 1.82 Dc	22.84 ± 1.15 Ec	97.9	< 0.01
	Bb + DE	78.15 ± 1.80 Ab	65.17 ± 1.78 Bb	61.44 ± 1.02 Bb	53.59 ± 1.58 Cb	40.62 ± 1.05 Db	87.1	< 0.01
	Bb + indoxacarb	87.69 ± 1.32 Aa	81.22 ± 2.08 Aa	73.03 ± 1.95 Ba	57.34 ± 0.93 Cb	53.24 ± 1.19 Ca	91.0	< 0.01
	DE + indoxacarb	95.22 ± 0.85 Aa	86.70 ± 1.34 Ba	77.82 ± 1.18 Ca	68.26 ± 2.01 Da	55.27 ± 1.54 Ea	117	< 0.01
	*F*	173	155	265	126	171		
	*P*	< 0.01	< 0.01	< 0.01	< 0.01	< 0.01		
*T. castaneum*	Bb	27.65 ± 1.98 Af	24.22 ± 2.53 ABd	19.45 ± 1.13 BCd	17.39 ± 0.83 BCd	13.31 ± 1.37 Cc	11.2	< 0.01
	DE	38.24 ± 1.80 Ae	31.73 ± 1.00 Bcd	24.57 ± 0.75 Ccd	23.56 ± 1.83 Ccd	20.12 ± 1.19 Cc	27.4	< 0.01
	Indoxacarb	45.73 ± 1.24 Ad	37.52 ± 1.66 Ac	29.35 ± 1.71 Cc	25.57 ± 1.97 Cc	17.40 ± 1.13 Dc	47.4	< 0.01
	Bb + DE	67.21 ± 2.16 Ac	56.28 ± 2.77 Bb	52.92 ± 1.37 BCb	46.40 ± 1.18 Cb	34.11 ± 2.23 Db	36.4	< 0.01
	Bb + indoxacarb	76.43 ± 1.41 Ab	72.37 ± 2.29 ABa	64.13 ± 2.04 Ba	48.46 ± 1.38 Cb	39.59 ± 0.74 Cab	47.3	< 0.01
	DE + indoxacarb	84.31 ± 1.52 Aa	75.08 ± 1.43 ABa	68.27 ± 1.90 BCa	64.86 ± 2.82 Ca	43.67 ± 3.06 Da	45.0	< 0.01
	*F*	173	111	102	105	49.6		
	*P*	< 0.01	< 0.01	< 0.01	< 0.01	< 0.01		
*T. granarium*	Bb	21.16 ± 1.81 Ae	17.41 ± 1.40 ABd	13.28 ± 1.71 BCc	10.23 ± 2.35 BCc	8.51 ± 1.42 Cc	8.55	< 0.01
	DE	25.59 ± 3.10 Ae	23.54 ± 2.55 Acd	18.75 ± 2.01 ABc	16.73 ± 1.15 ABc	12.28 ± 1.28 Bc	5.34	< 0.01
	Indoxacarb	36.86 ± 1.18 Ad	28.34 ± 2.36 Bc	15.69 ± 0.99 Cc	11.57 ± 1.86 CDc	7.20 ± 1.20 Dc	56.3	< 0.01
	Bb + DE	55.27 ± 2.81 Ac	51.18 ± 2.11 Ab	40.27 ± 1.23 Bb	29.35 ± 2.23 Cb	22.18 ± 2.18 Cb	41.6	< 0.01
	Bb + indoxacarb	67.56 ± 2.48 Ab	59.71 ± 1.60 Aab	43.32 ± 1.60 Bb	34.78 ± 2.10 Cab	27.63 ± 1.92 Cab	71.8	< 0.01
	DE + indoxacarb	78.83 ± 1.96 Aa	65.56 ± 2.89 Ba	54.62 ± 2.39 Ca	38.22 ± 0.98 Da	33.42 ± 1.56 Da	83.3	< 0.01
	*F*	92.0	84.4	100	43.0	42.1		
	*P*	< 0.01	< 0.01	< 0.01	< 0.01	< 0.01		
*R. dominica*	Bb	46.76 ± 1.12 Ae	43.67 ± 1.65 ABd	36.50 ± 2.34 BCc	28.67 ± 2.29 CDc	21.81 ± 2.47 Dc	25.6	< 0.01
	DE	55.27 ± 2.76 Ad	48.10 ± 1.92 ABcd	41.64 ± 1.29 BCc	35.14 ± 1.26 CDc	27.64 ± 1.71 Dc	33.1	< 0.01
	Indoxacarb	63.46 ± 1.85 Ac	52.21 ± 1.54 Bc	43.70 ± 2.04 Cc	31.18 ± 1.53 Cc	24.22 ± 2.04 Dc	66.8	< 0.01
	Bb + DE	86.67 ± 2.74 Ab	72.17 ± 1.78 Bb	65.20 ± 1.76 Bb	54.27 ± 1.83 Cb	48.46 ± 1.17 Cb	57.4	< 0.01
	Bb + indoxacarb	94.18 ± 1.23 Aab	86.69 ± 1.71 ABa	78.48 ± 2.74 Ba	66.58 ± 2.71 Ca	57.33 ± 1.24 Da	53.0	< 0.01
	DE + indoxacarb	100.00 ± 0.00 Aa	93.52 ± 2.19 Aa	84.37 ± 1.22 Ba	71.67 ± 1.91 Ca	63.80 ± 1.55 Da	90.4	< 0.01
	*F*	137	133	107	81.0	108		
	*P*	< 0.01	< 0.01	< 0.01	< 0.01	< 0.01		

For each species, within each treatment, means followed by the same uppercase letter are not significantly different; *df* = 4, 29, Tukey–Kramer (HSD) test at *P* = 0.05. For each species, within each period of storage, means followed by the same lowercase letter are not significantly different; *df* = 5, 35, Tukey–Kramer (HSD) test at *P* = 0.05

Concerning *T. granarium*, main effects and their interaction were significant when the individuals were exposed for 7 days (Table 5) and 14 days (Table 7). The *B. bassiana* alone and indoxacarb alone did not kill any *T. granarium* adult after 7 days of initial exposure, at the 120 days trial (Table 6). The most efficient treatment against *T. granarium* individuals was the DE + indoxacarb combination at all trials, 7 days post-exposure, with mortality ranging from 17.06 (120 days) to 46.40% (0 days). Indoxacarb caused the lowest mortality rates (7.20%) among all other treatments at the 120 days trial, 14 days post-exposure (Table 8). The combination of DE + indoxacarb achieved the highest mortalities at all trials, demonstrating a range from 78.83% at the 0 days trial and 33.42% at the 120 days trial.

As far as *R.* *dominica* is concerned, main effects and their interaction were significant after 7 days (Table 5) and 14 days (Table 7) of initial exposure. The DE + indoxacarb combination killed the most exposed individuals, reaching 72.37% mortality after 7 days of exposure, at the 0 days trial (Table 6). This combination was the most efficient against *R.* *dominica* adults at all trials. *Beauveria bassiana* alone caused the lowest mortality levels at all trials, ranging from 15.68 to 32.74%, 7 days post-exposure. Complete mortality was documented for the DE + indoxacarb combination, followed by the Bb + indoxacarb combination (94.18%) at the 0 days trial, 14 days post-exposure (Table 8). The *B. bassiana* alone treatments resulted to the lowest mortalities, at all trials (21.81–46.76%) after 14 days of exposure. The combination of DE + indoxacarb killed the most individuals at all trials with the 120 days trial exhibiting the lowest mortality of 63.80%.

Regarding progeny production, main effects and their interaction were significant for all the tested insect species (Table 9). The emergence of *C. ferrugineus*, *T. castaneum*, *T. granarium*, and *R. dominica* individuals at all treatments and trials was significantly lower than the control (Table 10). Among the treatments, *B. bassiana* alone was the least efficient and the DE + indoxacarb combination the most efficient at all trials, against all insect species. The lowest progeny production was recorded for *R.* *dominica*, not exceeding 13.66 individuals at the 0 days trial, on wheat treated with the DE + indoxacarb combination. The highest emergence was noted for the *B. bassiana* alone treatment, at the 120 days trial (107.67 *T. granarium* individuals per vial).

**Table 9:** ANOVA parameters of main effects and associated interaction for progeny production of *C. ferrugineus*, *T. castaneum*, *T. granarium*, and *R. dominica* individuals in laboratory persistence trials (total *df* = 209 for all species)

		*C. ferrugineus*		*T. castaneum*		*T. granarium*		*R. dominica*	
Source	*df*	*F*	*P*	*F*	*P*	*F*	*P*	*F*	*P*
Treatment	6	2488.64	< 0.01	2323.21	< 0.01	1299.97	< 0.01	1813.56	< 0.01
Period of storage	4	994.32	< 0.01	946.64	< 0.01	374.21	< 0.01	749.49	< 0.01
Treatment × period of storage	24	15.33	< 0.01	13.64	< 0.01	3.57	< 0.01	5.61	< 0.01

**Table 10:** Mean number (± SE) of *C. ferrugineus*, *T. castaneum*, *T. granarium*, and *R. dominica* individuals per vial following a 14-day exposure interval of parents on wheat treated with one dose of *B. bassiana* (Bb) (1 × 10^7^ conidia/kg wheat), one dose of DE (150 ppm), one dose of indoxacarb (5 ppm), their combinations (Bb + DE, Bb + indoxacarb, and DE + indoxacarb), and control in five laboratory trials carried out from 0 to 120 days after treatment

Species	Treatment	0 days	30 days	60 days	90 days	120 days	*F*	*P*
*C. ferrugineus*	Bb	51.26 ± 1.08 Eb	72.33 ± 0.78 Db	78.33 ± 1.83 Cb	85.23 ± 1.16 Bb	99.61 ± 1.11 Ab	203	< 0.01
	DE	48.63 ± 0.67 Eb	61.73 ± 1.33 Dc	74.30 ± 0.85 Cb	69.51 ± 0.21 Bd	84.30 ± 0.89 Ad	236	< 0.01
	Indoxacarb	37.73 ± 1.14 Ec	49.05 ± 1.94 Dd	62.80 ± 1.13 Cc	77.85 ± 0.42 Bc	91.65 ± 1.38 Ac	275	< 0.01
	Bb + DE	26.75 ± 1.34 Ed	38.11 ± 1.75 De	47.30 ± 1.50 Cd	56.20 ± 1.19 Be	64.23 ± 1.13 Ae	110	< 0.01
	Bb + indoxacarb	24.58 ± 0.58 Dd	32.50 ± 1.25 Cef	43.11 ± 2.14 Bd	48.33 ± 1.51 ABf	53.23 ± 0.85 Af	71.8	< 0.01
	DE + indoxacarb	18.20 ± 0.89 De	27.66 ± 0.79 Cf	35.28 ± 1.14 Be	37.35 ± 1.12 Bg	45.15 ± 0.86 Ag	110	< 0.01
	Control	87.86 ± 2.37 Da	111.27 ± 1.79 Ca	124.65 ± 0.73 Ba	130.67 ± 0.53 Ba	141.18 ± 2.35 Aa	139	< 0.01
	*F*	339	402	454	966	610		
	*P*	< 0.01	< 0.01	< 0.01	< 0.01	< 0.01		
*T. castaneum*	Bb	61.58 ± 2.23 Eb	68.91 ± 1.98 Db	83.48 ± 0.85 Cb	94.38 ± 0.74 Bb	102.38 ± 1.50 Ab	116	< 0.01
	DE	53.63 ± 1.60 Dc	62.83 ± 0.75 Cc	75.36 ± 0.96 Bc	87.58 ± 1.06 Ac	89.26 ± 1.23 Ac	178	< 0.01
	Indoxacarb	42.58 ± 1.30 Ed	48.26 ± 1.74 Dd	63.31 ± 0.95 Cd	75.40 ± 1.28 Bd	93.48 ± 0.70 Ac	272	< 0.01
	Bb + DE	33.65 ± 1.36 De	42.55 ± 0.46 Ce	47.70 ± 1.14 Ce	56.13 ± 1.20 Be	68.36 ± 1.65 Ad	116	< 0.01
	Bb + indoxacarb	27.21 ± 1.50 Def	34.23 ± 0.46 Cf	41.70 ± 1.85 Be	49.33 ± 1.21 Af	54.36 ± 1.17 Ae	68.9	< 0.01
	DE + indoxacarb	21.21 ± 1.04 Ef	28.31 ± 0.77 Dg	33.80 ± 1.11 Cf	38.31 ± 0.86 Bg	47.55 ± 1.03 Af	97.0	< 0.01
	Control	91.48 ± 1.51 Ea	102.22 ± 1.30 Da	115.78 ± 2.06 Ca	131.78 ± 1.07 Ba	142.30 ± 1.70 Aa	176	< 0.01
	*F*	242	434	442	873	598		
	*P*	< 0.01	< 0.01	< 0.01	< 0.01	< 0.01		
*T. granarium*	Bb	75.31 ± 1.24 Db	87.56 ± 1.75 Cb	92.80 ± 2.08 BCb	98.00 ± 1.23 Bb	107.67 ± 0.97 Ab	63.4	< 0.01
	DE	64.11 ± 1.05 Dc	81.20 ± 1.85 Cb	85.03 ± 1.17 BCb	93.51 ± 1.39 ABb	101.18 ± 2.35 Ab	36.5	< 0.01
	Indoxacarb	51.45 ± 1.04 Dd	65.38 ± 2.00 Cc	74.53 ± 0.92 Bc	76.58 ± 1.41 Bc	87.12 ± 1.03 Ac	42.8	< 0.01
	Bb + DE	38.45 ± 0.76 Ee	46.41 ± 1.36 Dd	61.05 ± 2.82 Cd	69.81 ± 1.46 Bc	78.25 ± 1.40 Ad	91.9	< 0.01
	Bb + indoxacarb	34.45 ± 1.94 Ce	38.60 ± 0.88 Cde	45.25 ± 2.42 Be	51.55 ± 0.93 Bd	64.76 ± 1.21 Ae	55.4	< 0.01
	DE + indoxacarb	26.36 ± 0.82 Df	31.30 ± 0.57 De	37.13 ± 0.87 Ce	43.88 ± 2.44 Be	52.23 ± 1.27 Af	55.6	< 0.01
	Control	101.28 ± 1.23 Da	115.43 ± 2.96 Ca	127.68 ± 2.11 Ba	136.15 ± 1.50 Ba	148.30 ± 0.93 Aa	66.7	< 0.01
	*F*	468	155	259	410	261		
	*P*	< 0.01	< 0.01	< 0.01	< 0.01	< 0.01		
*R. dominica*	Bb	44.03 ± 1.33 Eb	56.15 ± 1.22 Db	61.30 ± 0.63 Cb	75.58 ± 1.17 Bb	84.30 ± 0.89 Ab	216	< 0.01
	DE	41.06 ± 0.95 Db	45.71 ± 1.79 Dc	53.41 ± 1.06 Cc	64.21 ± 1.53 Bc	73.55 ± 1.26 Ac	96.5	< 0.01
	Indoxacarb	33.66 ± 1.12 Dc	43.45 ± 2.55 Cc	49.60 ± 1.13 Cc	68.79 ± 1.44 Bc	77.20 ± 1.09 Ac	131	< 0.01
	Bb + DE	25.30 ± 1.58 Cd	31.43 ± 2.70 BCd	36.41 ± 2.01 Bd	51.55 ± 0.77 Ad	56.83 ± 1.22 Ad	56.2	< 0.01
	Bb + indoxacarb	17.78 ± 1.26 De	26.16 ± 2.10 Cde	34.58 ± 1.92 Bd	43.28 ± 1.23 Ae	48.08 ± 1.32 Ae	58.6	< 0.01
	DE + indoxacarb	13.66 ± 1.60 Ce	18.35 ± 1.11 Ce	23.83 ± 1.10 Be	35.41 ± 1.30 Af	39.16 ± 0.74 Af	81.8	< 0.01
	Control	82.55 ± 1.07 Ea	97.30 ± 1.13 Da	106.32 ± 1.64 Ca	123.35 ± 0.77 Ba	128.88 ± 0.74 Aa	285	< 0.01
	*F*	316	187	353	576	776		
	*P*	< 0.01	< 0.01	< 0.01	< 0.01	< 0.01		

For each species, within each treatment, means followed by the same uppercase letter are not significantly different; *df* = 4, 29, Tukey–Kramer (HSD) test at *P* = 0.05. For each species, within each period of storage, means followed by the same lowercase letter are not significantly different; *df* = 6, 41, Tukey–Kramer (HSD) test at *P* = 0.05

## Discussion

The findings of our study suggest that all examined combinations resulted to higher mortality rates of *C. ferrugineus*, *T. granarium*, *R.* *dominica*, and *T. castaneum* adults than single applications. This phenomenon has been documented before with the binary combinations of *B. bassiana*, DE plus bitterbarkomycin (DEBBM), and imidacloprid causing higher mortality rates than each insecticide alone (Wakil and Schmitt 2015). Similarly, Hanif et al. (2022) showed that the 1.5 × 10^10^ *B. bassiana* conidia/kg + 50 mg/kg DEBBM or the 1.5 × 10^10^ *B. bassiana* conidia/kg grain + 150 mg DE /kg grain caused higher mortalities to *C. ferrugineus*, *R. dominica*, and *T. castaneum* adults than the entomopathogenic fungus and DE separately. We found that the most susceptible species was *R.* *dominica* followed by *C. ferrugineus*, *T. castaneum*, and *T. granarium*. More specifically, *R. dominica* was the only insect species that reached 100% mortality, when exposed on wheat treated with the DE + indoxacarb combination for 14 days. This phenomenon has been previously documented for *R. dominica* which was to be more susceptible than *T. castaneum* and *T. granarium*, when *B. bassiana* was combined with the phenyl pyrazole fipronil (Wakil et al. 2022). The knowledge of the order of stored-product insects’ susceptibility to certain insecticides, triggers the early applications so as to restrain further population growth and concomitant damage of the products (Colunga Garcia et al. 2013; Yemshanov et al. 2014; Douma et al. 2016; Kavallieratos et al. 2017b; Papanikolaou et al. 2018; Skourti et al. 2022). Whether this order remains the same when insect species coexist merits further investigation.

One of the most important findings is that the mortality levels of *T. castaneum* were moderate to high, ranging from 48.80 to 66.13% and from 63.03 to 87.42% for the tested combinations, after 7 and 14 days of exposure, respectively. *Tribolium castaneum* adults are hard to manage (Kavallieratos et al. 2021a, b, c, d); thus, their suppression is crucial because they consist reproductive vehicles (Arthur 1998a, b, 2008; Sehgal et al. 2013; Kavallieratos et al. 2017c, 2020). Since the mortality rates reached 87.42% at the DE + indoxacarb combination 14 days post-exposure, this insecticidal treatment could be considered for the effective management of *T. castaneum*.

The most efficient treatment against all tested species included the DE + indoxacarb combination. Even though the DE and the indoxacarb alone provided low to moderate mortalities and reduction of offspring emergence, their combination resulted to elevated efficacy at all laboratory and persistence bioassays. This could be attributed to the additive activity of the DE with indoxacarb, since DE causes cuticular lipid abrasion and/or absorption (Ebeling 1971; Korunic 2013; Shah and Khan 2014; Losic and Korunic 2018), rendering by this way the insects sensitive and prone to indoxacarb. Several studies have documented that DE enhanced the efficacy of synthetic insecticides (Wakil et al. [Bibr CR88][Bibr CR91], 2022; Wakil and Schmitt 2014; Machekano et al. 2017) or entomopathogenic fungi (Akbar et al. 2004; Lord 2005; Kavallieratos et al. 2006; Batta 2008; Sabbour et al. 2012; Ashraf et al. 2017; Wakil et al. 2021d). In the case of entomopathogenic fungi, DE increases the ability of entomopathogenic fungi conidia to attach the cuticle of the insect, and therefore advances its efficacy (Akbar et al. 2004; Shah and Khan 2014; Batta and Kavallieratos 2018). However, it should be noted that the combinations of DE and entomopathogenic fungi do not always lead to increased mortality of exposed insects, but they may result to detrimental effects (Vassilakos et al. 2006). One other important factor that determines the efficacy of the combination of *B. bassiana* and DE is commodity. For example, 1.5 × 10^10^ *B. bassiana* conidia/kg grain treated on maize and rice killed less *C. ferrugineus* than the combination of 1.5 × 10^8^ *B. bassiana* conidia/kg grain + 50 ppm DE, while on wheat the results were reversed (Wakil et al. 2021d).

Our results revealed that the *B. bassiana* + indoxacarb combination caused higher mortality rates than when each insecticide was applied separately. This issue could be attributed to the stress caused by the synthetic insecticide that weakens the insects’ immune system, allowing the entomopathogenic fungi to easily infect the insects (Hiromori and Nishigaki 2001). Numerous studies confirm that the efficacy of entomopathogenic fungi is enhanced by other insecticides (Ashraf et al. 2017; Wakil et al. 2012, 2022; Gad et al. 2020). However, the efficacy of a certain combination is affected by many factors (e.g., insect species, type of insecticide, entomopathogenic fungus species/isolate) that should be taken into consideration before a large-scale treatment takes place (Batta and Kavallieratos 2018).

The efficacy of *B. bassiana* was the least effective at the laboratory and persistence mortality bioassays but provided significant progeny reduction compared to control. The low efficacy could be the result of different fungal isolates (Moino et al. 1998; Mahdneshin et al. 2009; Wakil et al. 2021b). For example, among 7 *B. bassiana* Pakistani strains, there was considerable differences in the mortality rates of *R. dominica* (43.1–78.9%), *S. granarius* (41.2–72.2%), *T. castaneum* (36.2–60.7%), and *T. granarium* (23.4–43.1%) 15 days post-exposure (Wakil et al. 2021b).

The persistence efficacy of the tested combinations followed similar pattern as the laboratory bioassays. *Beauveria bassiana* provided the lowest mortalities and reduction of progeny, while the combination DE + indoxacarb the highest, against all species. All treatments lost gradually (from 0 to 120 days) their effectiveness to kill the tested insect species and suppress the offspring emergence. Each combination has residual efficacy that results to long term protection. In a previous study, Miliordos et al. (2017) found that indoxacarb alone exhibited elevated effectiveness for 180 days against *S. oryzae* and *R.* *dominica* on maize and wheat. Wakil and Schmitt (2015) documented that the dual combinations of DEBBM, *B. bassiana*, and imidacloprid protected effectively wheat from *C. ferrugineus*, *R.* *dominica*, *T. castaneum*, and *L. paeta*, for 6 months. In a recent study, Wakil et al. (2021c) proved that the combinations of DEBBM + *B. bassiana* or imidacloprid provided adequate protection of stored wheat when it was infested with *C. ferrugineus*, *R.* *dominica*, *T. castaneum*, and *L. paeta* for 180 days.

To conclude, this study suggests that *B. bassiana*, DE, and indoxacarb combinations are effective tools for the management of *C. ferrugineus*, *T. castaneum*, *T. granarium*, and *R.* *dominica*, when applied to stored wheat. However, the tested species suffered different levels of mortality as follows: *R.* *dominica* > *C. ferrugineus* > *T. castaneum* > *T. granarium*, at both laboratory and persistence bioassays. More studies that will include more parameters, like additional species and their developmental stages, commodities, and abiotic factors, are needed to assess the potential effect of the above promising insecticidal combinations for a sufficient protection of commodities in storage facilities. Last but not least, apart from DEs, other inert dusts such as zeolites or wood ashes exhibit elevated insecticidal properties against major stored-product insects (Bohinc et al. 2018, 2020). Therefore, it would be interesting to further investigate their effectiveness in combination with *B. bassiana* and indoxacarb as additional armory to the perpetual fight with stored-product pests.

## Data Availability

The datasets used and/or analyzed during the current study are available from the corresponding author on reasonable request.
